# A Pilot Study on Bioaccumulation and Tissue Distribution of Mercury in Barn Swallow (*Hirundo rustica*)

**DOI:** 10.3390/toxics12030206

**Published:** 2024-03-08

**Authors:** Li Tian, Yujing Zhu, Ruiming Yu, Xiaobo Zheng

**Affiliations:** 1 Life Science and Technology School, Lingnan Normal University, Zhanjiang 524048, China; tianli2019@mail.bnu.edu.cn; 2 Maoming Branch, Guangdong Laboratory for Lingnan Modern Agriculture, College of Natural Resources and Environment, South China Agricultural University, Guangzhou 510642, China; 20232167015@stu.scau.edu.cn; 3 School of Global Public Health, New York University, New York, NY 10044, USA

**Keywords:** stable isotope, mercury, bird, tissue distribution

## Abstract

Although extensive research has been carried out on the occurrence of mercury (Hg) in biota, bioaccumulation and tissue distribution of Hg in songbirds have not been well characterized. In the present study, Hg was investigated in insects and barn swallows (*Hirundo rustica*) to explore the bioaccumulation characteristics of Hg. Hg in swallow feathers and tissues including muscle, liver, and bone was investigated to determine the tissue distribution of Hg. The concentrations of Hg were 1.39 ± 1.01 μg/g, 0.33 ± 0.09 μg/g, 0.47 ± 0.10 μg/g, and 0.23 ± 0.09 μg/g in feather, muscle, liver, and bone samples, respectively. The trophic magnification factor of Hg in swallows and insects was higher than 1. However, the Hg concentrations in swallow feathers were not significantly correlated with stable isotope values of carbon or nitrogen, which implies the complex food sources and exposure processes of Hg for swallows. Feathers had significantly higher concentrations of Hg than liver, muscle, and bone samples (*p* < 0.01 for all comparisons). Feather, muscle, bone, and other organs had fractions of 64.4 ± 11.9%, 6.07 ± 2.06%, 20.0 ± 8.19%, and 9.56 ± 2.96% in total body burden of Hg in swallows. Hg in feathers contributed more than half of Hg in the whole body for most swallow individuals. Swallows may efficiently eliminate Hg by molting, and the excretion flux of Hg and other contaminants via molting deserves more investigation.

## 1. Introduction

Mercury (Hg) is a widespread and persistent environmental contaminant, which poses significant risks to wildlife and also humans. Mercury acts as a neurotoxin and potentially brings significant risks to the health of wildlife at sufficient concentrations [1,2]. Hg exposure will exert various adverse effects, including low reproduction output, suppressed immune function, altered breeding activity, and reduced cognitive performance [1,2,3,4]. In the aquatic anaerobic environment, inorganic Hg can be converted to methyl mercury (MeHg) under microbial processes. MeHg is more bioavailable and bioaccumulative than inorganic Hg in organisms and is prone to transfer through food chains into organisms at high trophic levels [5,6].

Birds have long been recognized as biosentinels of environmental pollutants, especially persistent, bioaccumulative, and toxic chemicals like Hg [6]. Seabirds and raptors have been frequently investigated for Hg pollution because they occupy high trophic levels in local ecosystems and can accumulate considerable concentrations of Hg [7,8]. Songbirds are less studied for Hg but are recently becoming popular environmental indicators of Hg exposure and risk [9,10,11]. Songbirds are abundant and widely distributed in different regions. They also feed on a range of dietary items increasing their exposure to anthropogenic pollution [6]. Though songbirds inhabit terrestrial environments, it has been proved that Hg in wetlands can accumulate in aquatic invertebrates and be transferred to terrestrial predators (including songbirds) via the consumption of aquatic emergent insects [12,13,14]. Songbirds usually have a wide range of movements in various landscapes and can also ingest nutrients and contaminants from terrestrial and aquatic prey. Although it is well known that Hg biomagnifies in food chains, diverse food source complicates the biomagnification factors of Hg [12,15]. Dietary sources and habitat-specific foraging led to highly variable concentrations of MeHg, and the ranges of MeHg levels differed up to 283-fold in a songbird species from the same sampling site [15]. Insectivores with higher fractions of aquatic prey sources may have higher MeHg concentrations than those with less aquatic prey sources and similar trophic positions. Hence, it is critical to trace various food sources and trophic positions when evaluating Hg concentrations in riparian wildlife.

Internal tissues of birds, such as blood, eggs, muscle, and liver, are generally considered the most reliable indicators of Hg exposure in birds [1]. However, analysis of the internal tissues requires lethal methods, which is always contradictory to ethical regulations and conservation goals. Feathers have been considered useful non-lethal alternatives for monitoring environmental pollutants [16,17,18]. In addition, feathers are more chemically and physically stable. Thus, feathers are suitable biomonitoring tools that are non-invasive and easy to preserve for a long time. Although feathers have been commonly used for monitoring Hg in avian populations, the reliability of feathers as a sampling matrix has not been thoroughly assessed for many songbird species [10]. In some cases, such as nonmigratory species, body feathers show a strong correlation with internal tissues. A previous study in songbirds reported that feather growth reduced the concentrations of Hg in blood and Hg levels in blood rose after the completion of feather growth [17]. Moreover, a significant relationship has been observed between blood and feather samples in the songbird species, Carolina Wrens [9]. Although molt was probably the main mercury excretion pathway in birds to accelerate the rate of depuration of Hg from blood or other internal tissues, research suggested that Hg depuration from organs in some songbirds was not consistently affected by the rate of molt [2,19]. For example, in the contrast analysis of accelerated molt and gradual molt finches, the difference in Hg levels in internal tissues (including brain, muscle, liver, and kidney) was not significant [2]. These studies reported inconsistent relationships between tissue and feather Hg concentrations [9,20,21], indicating that feathers do not accurately represent recent Hg exposure in some bird species. In addition, the tissue distribution of Hg in feathers and different internal tissues including muscle, liver, and bone was scarcely reported in the literature [22] and still needs to be testified for songbird species.

In order to better understand the food chain transfer and tissue distribution of Hg in swallows, the present study aimed to (1) examine the concentrations of Hg in different tissues of swallows, including muscle, liver, bone, and feather; (2) explore the potential trophic transfer of Hg; (3) elucidate the tissue distribution of Hg in swallows, which can indicate if feather can serve as an indicator of Hg pollution for different internal tissues.

## 2. Materials and Methods

### 2.1. Sample Collection

Body feather samples were collected from dead barn swallows (Hirundo rustica, *n* = 22, including 4 adults and 18 juveniles) discovered in a village close to Zhanjiang Bay, South China (20°53′ E, 110°10′ N), in 2021. Mineral exploitation, shipyard activities, and agricultural activities caused pollution in that region. The occurrence of Hg has been reported in previous studies [23,24]. The study area is a typical agricultural area of the subtropical plain in South China dominated by vegetable fields, sugar cane fields, and paddy fields including C4 and C3 plants. The main crop is rice, which can be harvested two or three times every year. The livestock reared in villages includes buffalos and chickens.

Most dead birds (*n* = 15) were completely dry when found on the road or in grassland, so only feather samples were collected for analysis. The birds were local breeding populations and were sampled from April to August, which was the breeding season for the swallows in the studied site. The swallows were found dead on the road or in grassland, but we were not sure if the swallows were roadkill or if they were dead because of the harsh summer conditions [25]. The bodies of seven birds were fresh and dissected for analysis of Hg in tissues. The muscle, liver, and sternum samples were acquired from seven swallows with fresh body conditions. Body feathers were rinsed with ultrapure water and freeze-dried. All samples were wrapped in aluminum foil and kept in freezers at −20 °C before further analysis. The tissues of individual swallows were analyzed. Insect samples were collected by sweep nets during the day and light traps at night. Locust and dragonfly samples were collected during the day, while cricket and beetle samples were collected during both day and night time. Because of the small body size of insects, insect individuals belonging to cricket, locust, dragonfly, and beetle were combined as composites to fulfill the analysis mass of stable isotopes and Hg concentrations. A total of 44 crickets, 48 locusts, 27 dragonflies, and 30 beetles were captured. In order to determine the detection limit of Hg and stable isotope, the insect individuals were mixed as four cricket, four locust, three dragonfly, and five beetle composite samples. The subsamples from each composite sample were used for Hg.

### 2.2. Sample Extraction and Analysis

All samples were first freeze-dried, and approximately 0.05 g of the dry sample was accurately weighed. The dry samples were ground to powder finer than 0.5 mm. Then, the samples were dipped in 4 mL HNO_3_ (65%) overnight. Then, samples were added with 3 mL H_2_O_2_ (30%) and digested at 120 °C for 4 h. The digestive fluid was cooled to ambient temperature, transferred to a volumetric flask, and diluted to 10 mL for instrumental analysis. Hg was quantified by atomic fluorescence spectrometry (AFS-8510, Beijing Haiguang, Beijing, China). All data in this study were expressed in μg/g dry weight (dw).

### 2.3. Isotope Analysis and Calculations

The measurements of δ^13^C and δ^15^N were the same as in previous studies [12]. Approximately 0.5 mg of dry sample was placed in a tin capsule and analyzed by a Flash EA 112 series elemental analyzer coupled with a Finnigan MAT ConFlo III isotope ratio mass spectrometer. Stable isotope abundance was calculated as follows:

δX = (R_sample_/R_standard_ − 1) × 1000 (1)

where X represents ^13^C or ^15^N, and R_sample_/R_standard_ is the ^13^C/^12^C or ^15^N/^14^N ratio. The precisions were ±0.2‰ (2 SD) for δ^13^C and ±0.5‰ (2 SD) for δ^15^N.

### 2.4. Trophic Magnification Factor (TMF)

The trophic level (TL) was determined by the following equation:

TL = (δ^15^N_sample_ − δ ^15^N_primary consumer_)/TDF + 2 (2)

where δ^15^N_sample_ is the δ^15^N of a swallow or an insect sample, and δ^15^N_primary consumer_ is the δ^15^N value of the beetle in the present study. The trophic discrimination factor (TDF) is set as 3.4.

TMF was calculated as follows:

lg C_Hg_= a + b × TL (3)

TMF = 10^b^ (4)

where C_Hg_ is the Hg concentration of the barn swallow feather or the whole insect, a is the y-intercept (constant), and b is the slope. Statistical significance of Equation (3) is defined at *p* < 0.05.

### 2.5. QA/QC and Statistical Analysis

Quality control was carried out by analyzing blanks and standard reference materials. Two blank samples were included with each batch, and the concentrations of chemicals in blank samples were deducted from those in experimental samples. Recoveries of Hg in standard reference materials (GBW10018 (GSB-9) standard substance for chicken [26]) ranged from 85% to 110% with relative standard deviations (SDs) less than 15%. The limit of quantification (LOQ) of Hg was three times the standard deviation of Hg detected in blanks and was 0.001 μg/g dry weight (dw) in the present study.

All statistical analyses were performed by SPSS 22.0 (SPSS Inc., Chicago, IL, USA). Concentrations of Hg were log-transformed to obtain normal distribution. T-test was employed to test for the significance of differences between Hg levels in different groups of samples. Pearson correlation analysis was used to test the correlations between Hg levels in different groups of samples. Significance was set as *p* < 0.05.

## 3. Results

### 3.1. Hg in Swallow Feather

Hg was detected in all swallow samples with concentrations of 1.39 ± 1.01 μg/g in feathers. Hg concentrations in swallow feathers were lower than Hg in feathers of seabirds, such as penguins (1.88 μg/g, [27]) and petrels (19.7 μg/g, [28]). The results of swallows were also lower than the concentrations of Hg (1.95 ± 1.37 μg/g) in feathers of raptors from the Marin Headlands of California, USA [7]. It is reasonable to observe lower concentrations of Hg in insectivorous birds than in seabirds and raptors because of the high trophic positions of seabirds and raptors. Songbirds (such as *Yuhina gularis*, *Pycnonotus xanthorrhous*, and *Suthora nipalensis*) from Southwestern China [11] had comparable concentrations of Hg (1.66 ± 1.03 μg/g) with swallows in this study. In the present study, Hg concentrations in swallow individuals were highly variable, ranging from 0.30 to 4.34 μg/g. Though the swallows stayed in the breeding seasons from April to August in the studied site, it is possible that some swallows have different wintering sites. Thus, the exposure residue during the wintering season can bring uncertainty in Hg bioaccumulation. The swallows have different foraging strategies and therefore different exposure values of Hg. As there were only 22 samples, we did not explore the temporal trend in the five months by statistical analysis method. The concentrations of Hg in feathers from fresh (*n* = 7) and dry (*n* = 15) swallow bodies were compared by *t*-test, and no significant differences were observed in Hg concentrations in the two groups (*p* = 0.21). The feather samples are more stable than other bird tissues under ambient conditions. The results indicate that the body condition of swallows does not have a significant effect on Hg concentrations in feather samples. The age of swallows also makes it difficult to predict the contamination values. In this study, the feather samples were from juvenile and adult swallows. However, it is difficult to confirm their specific age and molting time. The effect of age on Hg concentrations in feathers requires further study in the future. And more notably, Ackerman et al. [1] suggested that if Hg concentrations in bird blood were greater than 0.2 μg/g, it can be considered to have risk. According to the following formula [1],

ln C_Blood Hg_= 0.673 × ln C_Bird Feather Hg_ − 1:673 (5)

where C_Blood Hg_ and C_Bird Feather Hg_ are concentrations of Hg in bird blood (μg/g wet weight) and bird feather (μg/g dry weight), respectively.

The threshold of 0.2 μg/g Hg in blood was converted to 1.09 μg/g Hg concentrations in bird feathers. It is worth noting that eleven out of twenty-two swallows had concentrations of Hg in feathers higher than the threshold of 1.09 μg/g Hg in feathers, which could pose risk deleterious effects to birds [1]. Most studies focused on the exposure of Hg in aquatic and riparian food webs with high Hg basal and have noted the high exposure risk of Hg in these organisms. The result of the present study suggests that insectivorous birds may also suffer from elevated Hg exposure risks, even though insectivorous birds like swallows inhabit low basal Hg input terrestrial ecosystems. Terrestrial organisms like insectivorous birds may take up a certain amount of Hg via aquatic prey.

### 3.2. Influences on Hg Pollution

**Figure 1:**
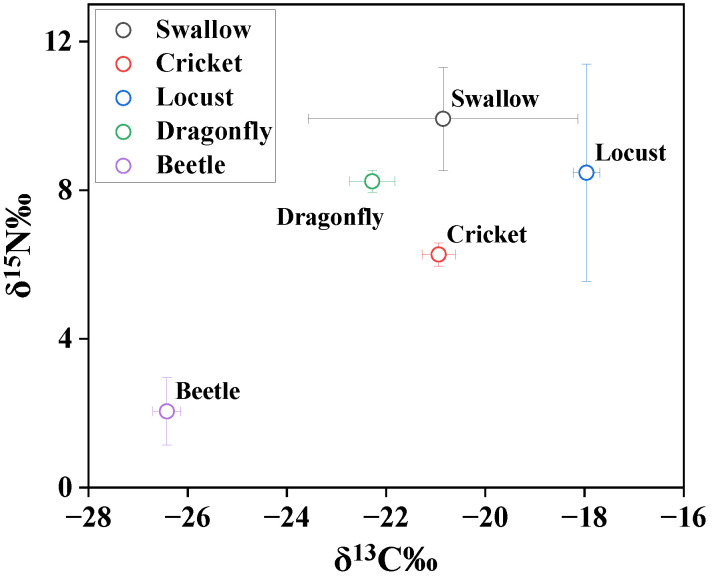
Stable isotope values in organisms.

In order to explore the food chain transfer of Hg from insects to swallows, Hg and stable isotopes of carbon and nitrogen were measured in insect species and swallow feathers (Table 1 and Figure 1). The stable carbon and nitrogen isotope signature indicates the diet source and trophic position, respectively, because δ^13^C increases by 0–1‰ and δ^15^N increases by 3–5‰ per trophic level [29,30]. The δ^13^C and δ^15^N values were −20.8 ± 2.72‰ and 9.92 ± 1.39‰ in swallow feathers, respectively (Figure 1). The δ^13^C values ranged from −18.0 ± 0.27‰ to −26.4 ± 0.28‰, and the δ^15^N values ranged from 2.05 ± 0.91‰ to 8.47 ± 2.92‰ in the insect species. The insect species had a broad δ^13^C range, indicating different carbon sources for insect species. δ^13^C in C3 plants ranged from 30.6‰ to 22.2‰, while C4 plants usually have δ^13^C values ranging from 20‰ to 10‰ [31]. The locust and beetle species seem to feed on C4 and C3 plants, respectively, while dragonflies and crickets are more likely to feed on mixed sources of C3 and C4. It was difficult to trace the feed sources of swallows by stable isotopes of carbon and nitrogen, as swallows showed δ^13^C values in the range of those in insects and higher δ^15^N values than those in insects. The investigated insects were all potential feeding sources for swallows.

**Figure 2:**
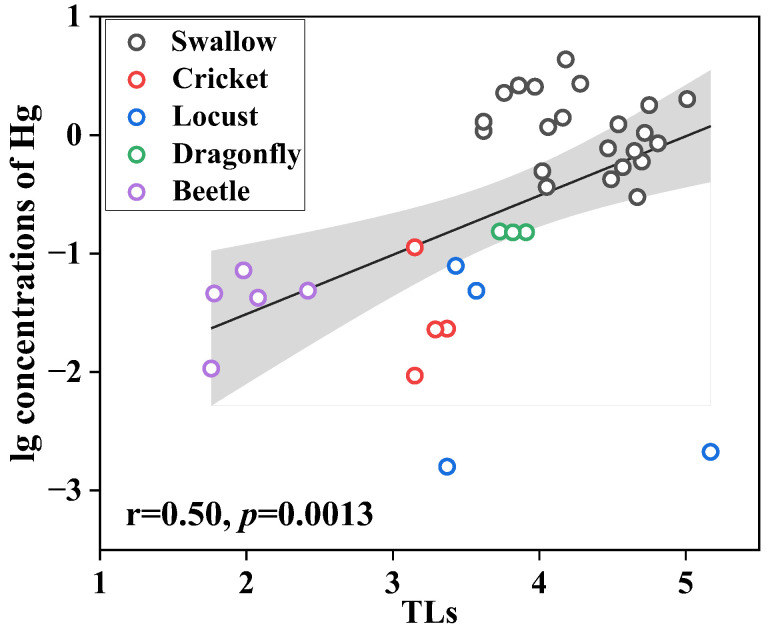
Relationships between log-transformed concentrations of Hg and trophic levels in samples. The black line and grey area mean the regression line of samples and the 95% confidence interval of regression line, respectively.

The mean concentrations of Hg were 0.03, 0.04, 0.04, and 0.15 μg/g in locusts, crickets, beetles, and dragonflies, respectively. Dragonflies have significantly higher Hg concentrations than other insect species (*p* < 0.05), which can be attributed to the aquatic habitat of dragonflies. Aquatic insects always exhibit greater exposure to Hg compared with terrestrial insects because of the microbial production of bioaccumulative MeHg in sediment [13]. Though the diet sources of swallows cannot be accurately estimated in this study, the insects and swallows were collected from the same village, which enabled discussions on the trophic transfer of Hg. Beetles had the lowest δ^15^N value (2.5 ± 0.91‰); therefore, it was deemed as the lowest TL (2.0 ± 0.27) herein. Among other species, crickets had the lowest TLs (3.24 ± 0.11), followed by dragonflies (3.82 ± 0.089), locusts (3.89 ± 0.086), and barn swallows (4.31 ± 0.41). Based on TL and δ^15^N values, the food web of insects–swallows was established in the present study. A significant positive correlation was found between trophic levels and log-transformed concentrations of Hg (*p* = 0.0013), as shown in Figure 2, which suggests a distinct transfer of Hg from lower to higher trophic levels.

The TMF of Hg in the present food web was calculated. The TMF (3.16) was greater than 1, indicating significant trophic magnification of Hg in the present food web. The TMF herein was slightly higher than the TMF of MeHg in a riparian food web (2.39), including insectivorous birds from another site in South China [12], and was similar to TMFs of Hg (2.64–3.02) in two marine food webs including seabirds from North China and Norway [8,32]. Cao et al. [33] reported the TMF value (1.69) of Hg from phytoplankton–invertebrate–fish food webs in Laizhou Bay, which was lower than the TMF of Hg in the present study, whereas a higher TMF value (5.7) of Hg from the primary producer–invertebrate–fish food webs in Poyang Lake was reported by Zhang et al. [34]. Variations in TMF values among different ecosystems might be due to factors such as food web structure, taxa groups, energy requirements of organisms, and temporal and spatial changes in sources of Hg for organisms [35]. Furthermore, due to the differences in the binding ability of Hg among tissues, different tissue types of organisms used in the analysis might also affect the TMF estimation. Previous research has reported that available binding sites in tissues for MeHg decreased with an increase in lipid contents [36]. Therefore, tissues containing a higher lipid content might be expected to have lower Hg concentrations [36]. However, only the feathers of swallows in the present study were used to estimate the TMF because of the limited swallow tissue samples. Further work is needed to evaluate the impact of the different tissue types on the TMF estimation in the future.

**Figure 3:**
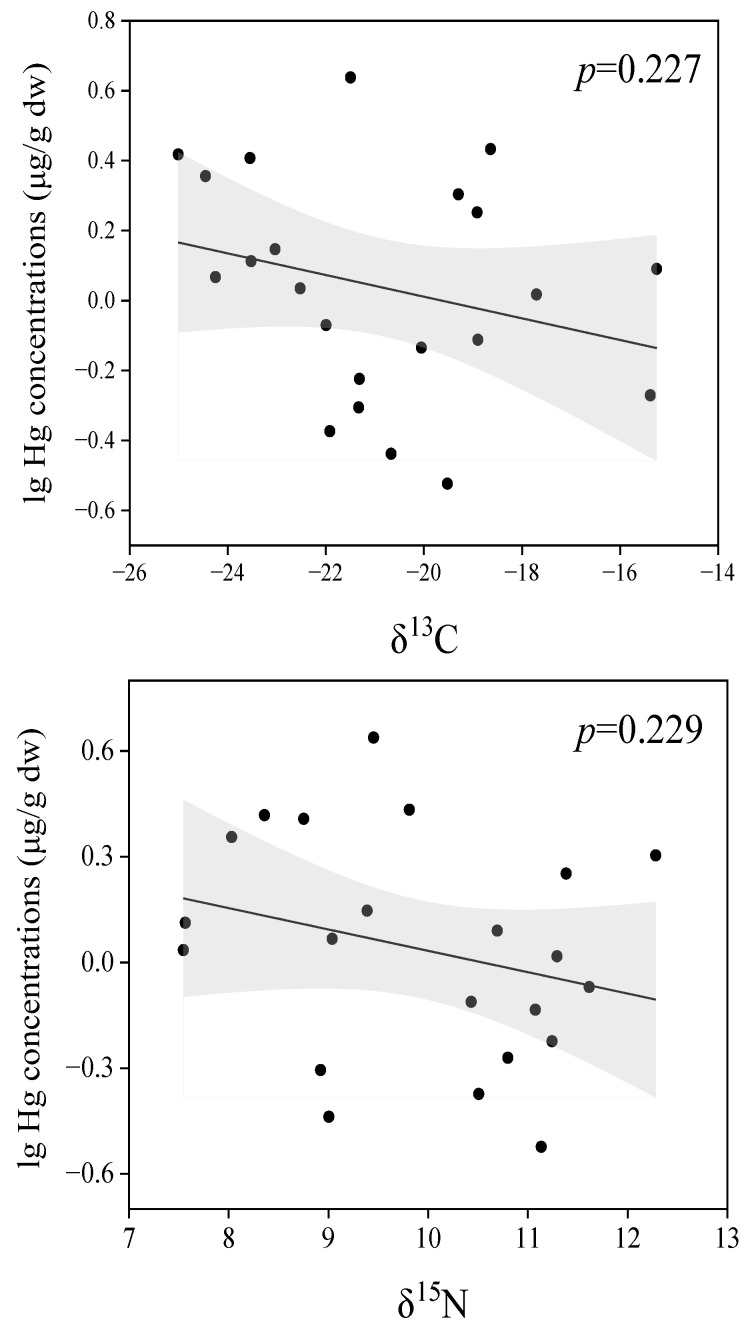
Relationships between Hg concentrations and stable isotope values in swallow feather samples. The black line and grey area mean the regression line of samples and the 95% confidence interval of regression line, respectively.

Correlations between stable isotope values and concentrations of Hg were tested for swallows, and no obvious relationship was observed between concentrations of Hg and δ^15^N or δ^13^C (Figure 3). It is demonstrated that Hg in bird feathers is mainly composed of MeHg, which is derived from trophic transfer in food chains [37]. However, concentrations of Hg in swallow feathers were not correlated with δ^15^N values in the present study, which indicates no obvious trophic transfer of Hg. The result could be explained by the different baseline values of δ^15^N. The diet sources are complex for the studied swallows based on the δ^13^C results. The different baseline values of δ^15^N in different insects and swallows affected the correlations between Hg concentrations and δ^15^N values. The basis of δ^15^N can also be affected by landscape types and agricultural activities in the studied region [38,39]. Wild creatures may receive organic and inorganic agricultural inputs of nitrogen from a broad area.

The high burden of Hg in certain terrestrial predators such as spiders [40], songbirds [11], and bats [41] has been frequently reported, which is mainly attributed to the contribution of aquatic food sources containing Hg. In adjacent food webs from South China, concentrations of Hg were significantly and positively correlated with δ^13^C rather than δ^15^N in terrestrial insects and birds [12]. Aquatic insects have been identified as a potential route for dietary metal exposure for terrestrial insectivores [11,12]. Higher values of δ^13^C indicate more proportions of aquatic prey in the total diet for songbirds, leading to high exposure levels of Hg. In contrast, no obvious relationship has been observed between the concentrations of Hg and δ^13^C values in swallows in the present study. Aside from the complex carbon sources for swallows, the concentrations and bioavailability of Hg are also suggested as important factors in the bioaccumulation of Hg. For instance, beetles have indigestible shells for birds and may have different bioavailability of Hg compared to other insects. In addition, the present study only collected three orders of insects, whereas swallows are known to forage on various species of insects. More ecology and contamination information on more insect species can help to elucidate the pollution risks for swallows.

### 3.3. Tissue Distribution of Hg in Swallows

**Figure 4:**
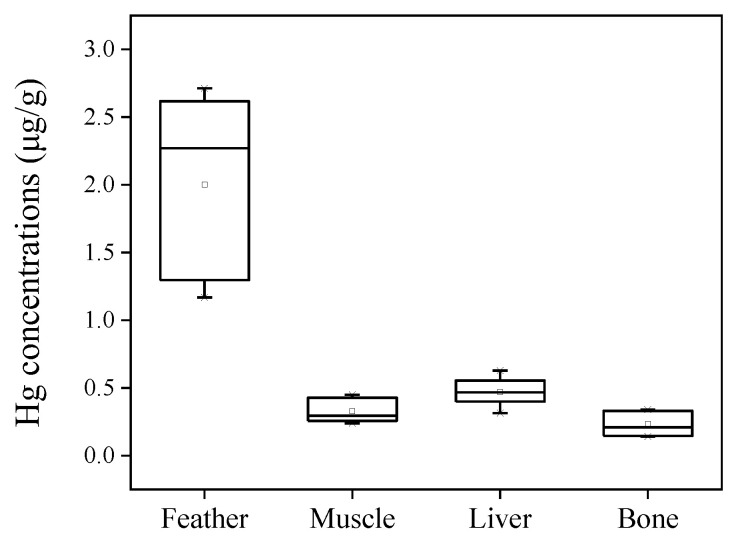
The ranges of Hg concentrations in paired swallow tissues. The boxes represent the range from the lower quartile to the upper quartile. The horizontal lines and the tiny squares in the boxes represent the median and average values, respectively.

Most studies have monitored pollutants in the feathers and internal tissues (including muscle, liver, and blood) of birds [6]. Feather was proposed as a non-invasive monitoring sample for birds. Some studies have tried to assess the relationships between Hg concentrations in feathers and internal doses in blood [9,16,17]. In the present study, the muscle, liver, and bone samples were available for seven swallows. The tissue distribution of Hg was then discussed. Hg was detected in all swallow tissues. The concentrations of Hg were 0.33 ± 0.09 μg/g, 0.47 ±0.10 μg/g, and 0.23 ± 0.09 μg/g in the muscle, liver, and bone samples, respectively (Figure 4). Feathers had significantly higher concentrations of Hg than internal tissues including muscle, liver, and bone (*p* < 0.01), whereas bones had the lowest Hg concentrations among all tissues (*p* < 0.05). Correlations between the concentrations of Hg in feathers and other tissues were assessed by Pearson correlation analysis, and no significant correlations were observed between feathers and muscles, livers, or bones (*p* > 0.05). Previous studies have reported that Hg concentrations were the highest in feathers and nails, followed by the liver and then the muscle, among tissues [21,42,43]. For instance, mean Hg concentrations in the thrush family (*Turdidae*) approximated a 13:3.57:1 ratio for feather, liver, and muscle tissues, respectively, as reported by Low et al. [44]. This trend was consistent with the findings in the present study.

**Figure 5:**
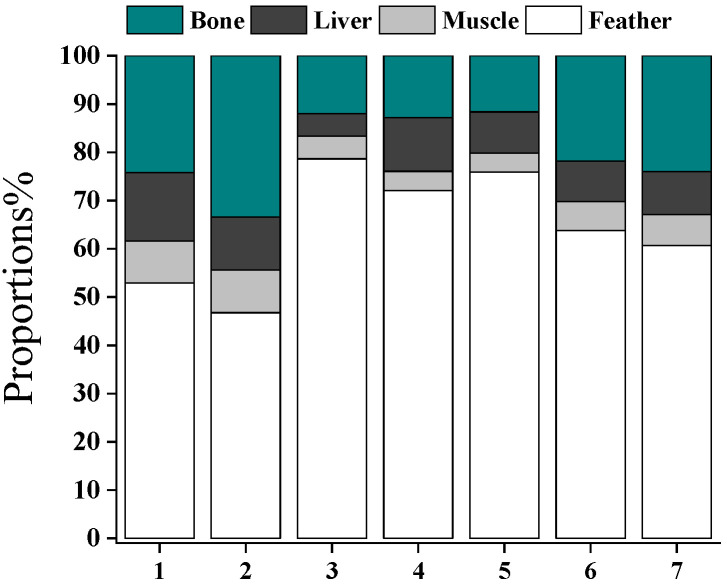
Proportions of Hg burden in swallow tissues. Each bar represents a swallow individual.

The tissue burden of Hg (g) in feathers, muscles, bones, and other organs was roughly estimated as concentrations of Hg multiplied by the weight of different tissues. The liver concentration of Hg was used to calculate the burden of Hg in all viscera organs. Feather, muscle, bone, and other organs had fractions of 64.4 ± 11.9%, 6.07 ± 2.06%, 20.0 ± 8.19%, and 9.56 ± 2.96% in the total body burden of Hg in swallows (Figure 5). Hg in feathers contributed more than half of Hg in all tissues for most swallow individuals, indicating that swallows can efficiently eliminate Hg via molting. Previous studies verified that variations in Hg concentrations existed between inner tissues and feathers, and the order of magnitude of higher Hg concentrations in feathers compared to inner tissues led to an overestimation of Hg burden in birds [44]. In comparison with other tissues, feathers deposited the largest proportion (i.e., the highest concentrations) of Hg originating from diet exposures. The results indicate that feathers may not be a suitable indicator of Hg contamination in internal tissues. However, Hg in feathers is still worth attention because feather molting seems to be an important excretion pathway of Hg for swallowing. As the chick gains mass, the pollution of Hg will dilute in the body burden. Thus, the newly growing feathers allocate Hg, and Hg concentrations in blood and other internal organs will decrease. The excretion flux of Hg and other contaminants via molting warrants more studies in comparison with contaminants in the eggs and feces of birds.

Overall, the present study performed a pilot investigation on the bioaccumulation and tissue distribution of Hg in swallows from a tropical region. There are some limitations in the present study. The swallow populations seem to have complex diet sources, which is indicated by the relatively wide range of δ^13^C and δ^15^N values. The manuscript collected limited swallow and insect samples, and the insect composites were analyzed. A more comprehensive survey on swallow diet composition and diet pollution is necessary to elucidate the exposure, bioaccumulation, and risk of Hg and more contaminants in swallows. It should be noted that the survival and reproductive success of swallows in the studied region were relatively lower than in other temperate regions according to our recent observations [25]. The harsh summer conditions in the studied tropical region such as high temperatures, high humidity, rainstorms, and typhoons were suspected to pose negative effects on swallows. However, the toxicological risks of Hg and other contaminants for the local bird populations are not well understood. More research is required to fill the knowledge gap on swallows, which can serve as a useful bioindicator of environmental and ecological conditions.

## 4. Conclusions

The present study measured Hg in swallow tissues and insect species. However, the Hg concentrations were highly variable in swallow feathers and were not significantly correlated with δ^13^C or δ^15^N values, which may be attributed to the pollution levels and bioavailability of Hg in complex diet sources of swallows. Feathers had the highest concentrations of Hg, while bones had the lowest concentrations of Hg among the three tissue types of swallow samples. The estimation of the body burden of Hg shows that feathers are the primary deposit for Hg, with fractions of 64.4 ± 11.9% in the total body burden of Hg in swallows. These results indicate that swallows can efficiently eliminate Hg via molting, which should be of interest in the evaluation of exposure and risk of Hg for swallows and other bird species, and the excretion flux of Hg via molting warrants further study.

## Figures and Tables

**Table 1:** Sample information and Hg concentrations (μg/g dw).

English Name	N	δ^13^C‰	δ^15^N‰	Hg
Barn Swallow (*Hirundo rustica*)				
Feather	22	−20.85 ± 2.72	9.92 ± 1.39	1.39 ± 1.01
Muscle	7			0.33 ± 0.09
Liver	7			0.47 ± 0.10
Bone	7			0.23 ± 0.09
Insects				
Cricket	4	−20.94 ± 0.33	6.27 ± 0.31	0.04 ± 0.05
Locust	4	−17.96 ± 0.27	8.47 ± 2.92	0.03 ± 0.04
Dragonfly	3	−22.28 ± 0.46	8.24 ± 0.3	0.15 ± 0.00
Beetle	5	−26.42 ± 0.28	2.05 ± 0.91	0.04 ± 0.02

## Data Availability

Data are contained within the article.
